# Licentiate Blich’s Trial of the Cold Water System

**Published:** 1842-08-27

**Authors:** 

**Affiliations:** Christiana


The only rational attempt at testing the therapeutic merits, if such there be, of hydropathy, is one by M. Blich, of Christiana. A powerful alterative, we have every reason to believe it is, but accompanied with so many most inconvenient and dangerous consequences, that we are confident no one in his senses would ever submit to the pure and absolute treatment, after the novelty had passed. As eclectics, however, we think it desirable that some one should find the pure grains of wheat in the mass of chaff, and M. Blich has set about the task in a sensible and very able way.

*Trial of the Cold Water System.* By LICENTIATE BLICH, of Christiana.—The author tried the plan in sixty-five cases, all chronic, in the majority of which a decided dyscrasia was present. The violent and daily perspiration, which is the special characteristic of the Grafenberg water-cure, was employed by the author, as a general rule, only when the dyscrasia was very manifest. The following is his general method of treatment. First comes a preparatory course, lasting from three to six days; it consists in washing the whole body with cold water in the morning, and then walking about in the open air. At the expiration of this period, the patient is stripped, wrapped up pretty tightly in blankets as far as the neck, and round it, and then covered with a large counterpane. The head is enveloped in a cloth, which projects over the face and cheeks, so as to leave the mouth and nose free. This having been done, in most instances a copious perspiration breaks out, within two or three hours, or frequently sooner; this is kept up from two to four hours, while the patient drinks cold water from time to time, to quench his thirst, and promote exhalation. The patients are now taken out of the blankets; and to avoid the trouble of having a bath in the room, the author places them in a flat empty tub, and has them sprinkled with one or two pails of water. The temperature of the water varies from 8° to 18° of Reaumur (50° to 72½° of Fahrenheit,) according to the greater or less sensibility of the patient; and while this is going on, he rubs himself briskly with his hands, to diminish the sudden feeling of cold. The patient then dresses, and in case the shower bath has made him too cold, drinks a glass of cold water, takes a short walk in the open air, and then breakfasts on bread and butter and cold milk. Another walk is taken before noon, and a couple more in the afternoon. Two or three measures of cold water are drunk daily, particularly before and during walking; and according to circumstances, clysters, injections, sitting and other local baths, fomentations or douches (all of cold water,) are employed. The diet is nourishing, but not stimulating, and warm drink or food is not allowed. The patients usually followed their ordinary occupation during the course, but with moderation.

In this manner the author observed the chief points of the Grafenberg system, namely, by introducing a quantity of water into the system, allowing it to pass through, and be secreted from it again, together with different morbid materials, (or whatever they may be called,) by the increased and critical evaporation from the skin, without any consequent relaxation of the cutaneous surface, or weakening of the restorative power. Before the author proceeds to give a report of the individual cases, he premises some remarks on the various points of the treatment, as far as his observations differ from those hitherto made, or may serve as a supplement to them.

*Sweating* was employed in the majority of cases, but only once a day, with the exception of 16 cases*; namely, in eight patients who had nervous rheumatic pains, and exhausting perspiration; one with copious hectic morning sweats; two with varicose swellings: one with disease of the knee-joint; one with chronic vomiting; and three with bronchitis. It was more or less

* The sentence is so obscurely expressed, as to leave it doubtful whether these sixteen cases were sweated more than once a day, or not at all; but it is clear from the rest of the paper that the former is intended.—*Translator's Note.*

difficult to produce perspiration. In some instances it was necessary to cover the face, before perspiration could be called forth; in one patient only, who was suffering from ascites, it was impossible to produce it at all. One of the most constant inconveniences from the continuance of the sudorific treatment is obstinate costiveness, which, however, often yielded in part to the cold clysters, and as the treatment went on, got right of itself.

The water drunk seldom exceeded from from three to four measures daily, but was often less; it showed its benefits more decidedly, and with special rapidity in chronic irritation of the stomach accompanied by vomiting, both when used alone, and when combined with sweating. The hearty appetite which many patients gain during the course, the satisfying which does not always agree with them, is also partly diminished by the liberal use of cold water at meals.

If too much water is drunk at the beginning of the course, the urine is often increased at the expense of the perspiration; and those patients who are unable to take sufficient exercise, feel uncomfortably cold, and take a disgust to the water. The douche was only employed in a few cases, chiefly local ones, where there was atrophy, or imperfect paralysis of a limb. Its effect was excellent, and it seems to have brought out local and critical eruptions.

The sitting-bath was applied chiefly in diseases of the abdomen, and affections of the urinary passages and genital system; but in a few cases it produced headache and obstinate rheumatic ophthalmia.

The author observed a true febrile crisis only once. It occurred on the seventh day of the treatment of a diseased knee-joint, was combined with severe headache, violent pain over the whole body, which lasted two or three days, and ended after a critical perspiration and vomiting. In three cases only the author observed considerable diarrhœa; in the rest, merely the usual cutaneous eruptions, particularly on the places where the fomentations were applied. These were absent in very few cases.

The majority of the diseases which the author treated in this way were of a rheumatic type; either rheumatic neuralgiæ, or other diseases in which rheumatism appeared to play the principal part. Thirteen were cases of common rheumatic neuralgiæ; eleven being men, and two women. In seven of the women the pains were chiefly in the limbs: in two there was cardialgia; one suffered from hemicrania, and one from neuralgia in the breast, with gout in the hip-joint. Twelve of these cases were cured or relieved for a time; in two, the method failed completely.

Another disease, in the treatment of which the author promised himself much from the Priessnitzian method, and the more so because baths were long considered a principal remedy in its cure, was scrofula. There was no disease, however, in which the results were so unfavourable; for of six patients, three died, two were relieved, and only one cured, and even in this instance it is not known whether the disease has returned.

Four cases of inveterate syphilis were treated. In two there was sore-throat, accompanied in one case by condylomata; in a third there were sores on the tongue; and all three had had several relapses after the use of mercury, sarsaparilla, and guaiacum. In one patient the treatment was pursued for five months, and effected a perfect cure without relapse. The second and third were cured of the local affections in a couple of months; but one became tired of the treatment, and the other was obliged to go away, and therefore discontinue the treatment. The author has heard that the symptoms returned afterwards in both cases.

In the fourth case, which was cured, the patient, besides having been subjected to Dzondi's course of corrosive sublimate, had used assafœtida and decoction of sarsaparilla, besides local remedies. In this instance the perspiration had a frightful fetor—the only example among the author's cases.

Of two cases of chronic disease of the skin, one was a patient suffering from eczema impetiginoïdes, who is still under treatment. The other was afflicted with psoriasis inveterata of several years' standing, which during the last year had spread over the whole body, except the palms of the hands and the soles of the feet. He used a variety of remedies without the smallest benefit. The water-system, which at first excited considerable diarrhœa and perspiration, produced no benefit in six weeks; so that the patient could no longer be persuaded to continue the course, which had caused him so much inconvenience and pain. After the nitrate of silver had also been tried in vain, he was rubbed for some days with the common tar ointment. The cuticle now fell off in large pieces, and under it was formed a fresh one, without scales, except in a few places; and in those, too, the scales vanished in a few days, after the energetic employment of sweating. A year has now passed, and there has been no relapse.

This case is one of those which have induced the author to think that a combination of the water plan with local remedies is beneficial in many cases, and effects an alteration in the structure of the diseased organ; while the new treatment alone could only do this slowly, and that indirectly, by removing the dyscrasia.

*Diseases of the mucous membranes, and blenorrhœa.*—The author treated twelve cases of this class, where, for the most part, the intestinal canal, or the trachea and its ramifications, were affected. Among these, the best results occurred where there was chronic inflammation of the mucous coats of the stomach, for of five patients four got well. The fifth (uncured) was affected at the same time with scirrhus of the stomach.

The treatment of chronic inflammations of the trachea and bronchi was less fortunate. There was one case of laryngitis, and four of bronchitis. The case of laryngitis is still under treatment. Of the four cases of bronchitis two were complicated with crude tubercles. One of these was cured by cold washing and frictions, so that a cough which had lasted several months was cured, and the patient considered himself well. The other, whose cough was accompanied by considerable hæmoptysis, became worse, has hectic fever, and will hardly live through the summer. The third suffered at the same time from emphysema of the lungs, and constant night-attacks of asthma, and is also uncured, although the mucous rattle was much lessened during his two months' course. The fourth still continues the course, but with little effect.

*Blenorrhœa of the vagina*, which was combined with several of the previous cases, was, in general, cured or considerably relieved during the course.

*Diseases of the serous membranes, and serous effusions.*—The author treated one case of arachnitis spinalis accompanied by partial paraplegia, besides a case where there was ostitis, (osteitis ?), two of hydrops ovarii, one of anasarca, and two where there was effusion into the joints; but as these last ones cannot be considered separately, they are mentioned under other heads.

*Diseases of the abdominal organs.*—The author can say nothing positive about these maladies, except the affections of the mucous membranes above mentioned; though the treatment of such cases is said to have been universally successful at Grafenberg.

*Organic diseases of the abdomen.*—Besides the case of scirrhus of the stomach, and one of hypertrophy of the liver, which were not affected by the course, the author treated a patient suffering from infarctus of the liver and spleen, and one suffering from ascites, but both only for a short time, and without advantage.

*Diseases of the brain and of the thoracic organs.*—In addition to the cases already mentioned, there was one of hypertrophy of the heart with induration of the aortic valves, and œdematous swelling of the feet; but the treatment was of no avail.

*Diseases of the uterus and ovaries* were treated with greater success. One patient, with a fibrous tumor of the uterus, was bed-ridden, and emaciated in the highest degree. The water treatment perfectly restored her general health, but the tumor remained the same. Another woman was suffering from polypous excrescences in the os uteri, as well as a long train of hysterical symptoms. The treatment had no effect upon the polypi, but most materially relieved the hysteric rheumatalgia.

There were three cases of ovarian dropsy, which in two instances were combined with scirrhus. In these two there was no perceptible improvement; and in the third case the treatment was not able to effect much in five weeks.

The treatment was employed for two months against a scirrhus of the breast; it removed the pain for a time, but the swelling remains the same.

*Chronic induration of the parotid gland, with fistulous sores.*—This was probably a case of the enchondroma described by J. Miller. In a couple of months the swelling was considerably diminished, but the patient could not be induced to continue the course.

*Chronic inflammations of the joints, and affections of the bones.*—These were the cases in which the treatment was most efficacious. There were six cases of diseased joints; four where the malady was in the knee, one in the hip, and one in the shoulder. Four of these patients were cured, and one relieved, who is still under treatment.

In one case, the disease was in the knee-joint, and there was a considerable effusion of synovial fluid, accompanied by constant pain, and a wasting of the whole extremity. The patient, a woman 40 years old, had been treated for two years in the usual way with leeches, moxa, and a whole series of derivative and resolvent remedies, but without advantage. The water treatment continued for a year dispersed the accumulated fluid; and for several months before the end of the term she was able to walk without a stick. In a few days after its commencement, the course produced a strong reactional fever, and the knee, the lower part of the thigh, and the calf, were covered during the whole time with pimples, pustules, and boils.

*Caries of the bones, with chronic fistulous ulcers.*—Four cases were treated of this disease. One was a caries of the vertebræ, one of the mastoid process, and two of the femur. Two are still under treatment, of whom one is considerably relieved. The other two are reported as cured; but the author thinks it right to state that he has not spoken to them, since they discontinued the treatment.

Lastly, Licentiate Blich treated a varicose ulcer of the leg of sixteen years' standing, and cured it perfectly in half a year, though the varicose dilatation of the veins remains as before.

A case of erectile swelling on the arm has long resisted the treatment, which is still continued. On the whole, of 65 patients, 20 have been cured; 14 have discontinued the treatment without being cured; 15 were not cured, of whom five died, and three of these probably in consequence of the treatment; and 15 are continuing the treatment, with more or less hope of a favourable result. In one case the result is not known. The length of the treatment varied from three days to a year; the average was ten weeks and seven-eighths. It succeeded best in irritation of the mucous membrane of the stomach, and inflammation of the joints; and worst in glandular diseases with tubercular formations in the nobler internal organs.—*London Med. Gazette*, June 10, 1842, *Abridged from Schmidt's Jahr.*

